# ﻿Rediscovery of a lost semi-aquatic Leaf Beetle in the Hula Valley, Israel (Coleoptera, Chrysomelidae, Donaciinae)

**DOI:** 10.3897/zookeys.1177.101498

**Published:** 2023-08-30

**Authors:** David G. Furth, Matteo Montagna, Giulia Magoga

**Affiliations:** 1 Department of Entomology, Smithsonian Institution, Washington, D.C., USA; 2 Steinhardt Museum of Natural History, Tel Aviv University, Israel; 3 Department of Agricultural Sciences, University of Naples Federico II, Via Università 100, 80055 Portici, Italy; 4 Interuniversity Center for Studies on Bioinspired Agro-Environmental Technology (BAT Center), University of Naples Federico II, Via Università 100, 80055 Naples, Italy; 5 Department of Agricultural and Environmental Sciences -Production, Landscape, Agroenergy, University of Milan, Via Celoria 2, 20133, Milan, Italy

**Keywords:** conservation, DNA taxonomy, Hula Lake and Swamps, land reclamation/restoration, morphology, reed beetles, wetland drainage

## Abstract

Between 1951–1958, most of the Hula Lake and its surrounding swamps in the Upper Jordan River (Rift) Valley of Israel were drained with the supposed purposes to eliminate malaria and to reclaim land for agriculture; both reasons later proved to be unnecessary decisions. With the paucity of biological knowledge of the Hula region, especially its aquatic invertebrates, accurate assessment of the environmental damage from this drainage is still being realized. Based on natural history museum collection specimen records, the pre-drainage presence of some aquatic insect species has been verified. Among these was *Donaciabicolora*, a member of a semi-aquatic subfamily (Donaciinae) of Leaf Beetles (Chrysomelidae) and whose Israeli populations were thought to have gone extinct because of the drainage of the Hula and other locations. Recently this species was rediscovered in two populations. However, the molecular identification of two of these recently collected specimens from one population revealed that the identity of this species is actually *Donaciasimplex*. In this work, the re-discovery of this species is detailed, and its conservation importance discussed.

## ﻿Introduction

**Figure 1.**
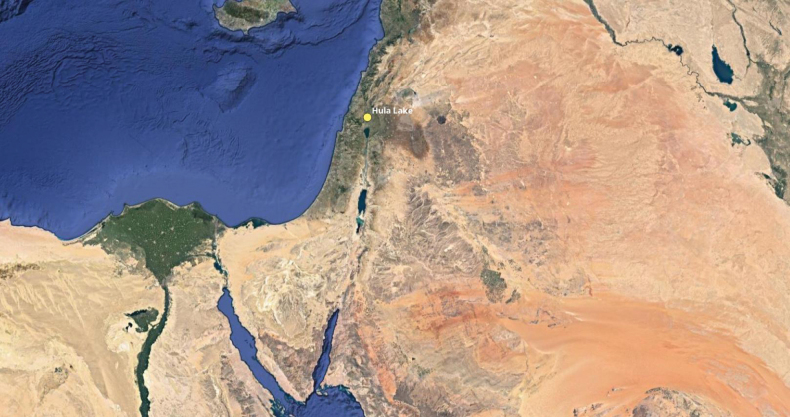
Map of Middle East (partial) with the Hula Lake Preserve indicated.

**Figure 2.**
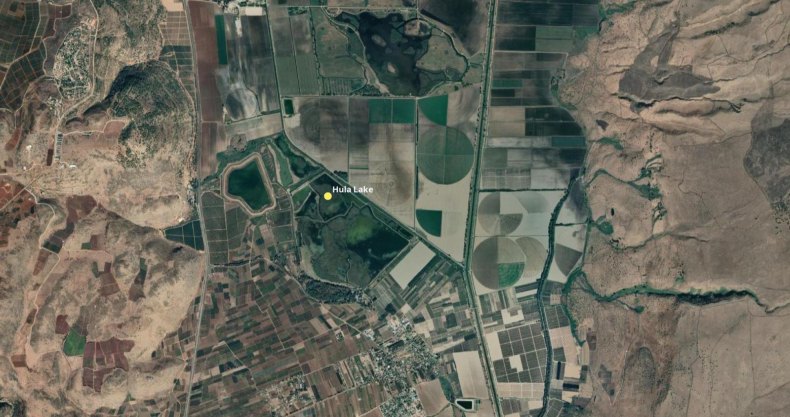
Map of current Hula Lake Preserve surrounded by agricultural fields.

The Hula (sometimes spelled Huleh) Lake and Swamps (Figs 1, 2) are considered as the “limnological lungs” of the Jordan River, i.e., the sources of this important river (FD Por, in Dimentman et al. 1992). The area has a long history of thousands of years in the ancient literature such as those by Pharoah Amenhotep IV (14^th^ Century BC), Josephus Flavius (1^st^ Century AD) and others (Dimentman et al. 1992). Even in the Talmud (1^st^ Century AD) it is referred to as the Merom; in limnological studies in the Hula Valley by G. Evelyn Hutchinson and colleagues as the “Waters of the Merom” (Dimentman et al. 1992). The first true natural history studies of the Hula were done by Tristram between 1864 and 1884 (Tristram 1884), mostly a survey of vertebrates and a few other groups like molluscs, a few water beetles, dragonflies, and other invertebrates, as well as the flora (Dimentman et al. 1992). When the idea of draining the Hula was first proposed in the early 20^th^ Century, the 1935 Percy Sladen Expedition to the Hula was initiated and surveyed mostly plants, some vertebrates, and a few invertebrate groups (Paz 1976). Other pre-drainage floral information can be found in Jones (1940). Part of the reason for this paucity of knowledge of the fauna and flora was because the Hula area was considered too dense for explorers to penetrate, as well as the presence of Malaria (Dimentman et al. 1992).

Previously the senior author (1977) reported that Israeli populations of *Donaciabicolora* Zschach, 1788 (Coleoptera, Chrysomelidae, Donaciinae) and *Galerucellanymphaeae* (Linnaeus, 1758) (Chrysomelidae: Galerucinae) were apparently extinct there due to the drainage of the Hula Lake and Swamps between 1951 and 1958. This drainage apparently caused the local extinction of various plants and animals (Furth 1977, 1993), including the known food plant of *D.bicolora*. Also mentioned in these publications was the previously reported *D.thalassina* Germar, 1811 (= *Donaciamarginata* Hoppe, 1795) and that both these species were known to feed on the same or congeneric food plants, *Sparganiumerectum* Linnaeus 1753 [Typhaceae].

This study is an update of the long history of awareness of fauna and flora that were devastated or even extirpated from the Hula Lake and Swamps effected by the drainage of these areas between 1951–1958. This drainage was conducted because of ideas that this would allow arable land reclamation and would eradicate a significant malaria epidemic there or, as has been said, to “sanitate the malaria infested and evil marshes and to turn them over to healthy agriculture” (Dimentman et al. 1992). But after this drainage it was discovered that the reasons were mistaken. Subsequently the government attempted to restore the Hula Lake and Swamps by re-inundating these areas with new water sources. The Hula Lake and Swamps became the first national park in Israel in 1964 and now is both a World Heritage site and a RAMSAR site, rich in wetland habitats and therefore a special place to view migrating birds (Fig. 2).

The biogeography of the Hula is rather unique because it is the northern limit of many Afrotropical species and the southern limit for Palearctic species. For example, Tristam (1884) stated that ~ 30% of the mammal species of the Hula were Afrotropical species and Kugler and Wool (1968) thought ~ 50% of the midge (Diptera: Chironomidae) species were Afrotropical. Other more recent examples can be found in Dumont (1991) for Odonata and Yanai et al. (2020) for Ephemeroptera. One of the dominant plants in the Hula is Papyrus (*Cyperuspapyrus* Linnaeus, Cyperaceae), an Afrotropical species; the Hula is the only place outside Africa where this plant is found naturally. Similarly, several species of *Tilapia* (Cichlidae) are found in the Hula, the only place they are known outside Africa.

In addition to *D.bicolora*, several animal species were thought to have been extirpated by the drainage of the Hula, such as the endemic Hula Painted Frog (*Latonianigriventer* (Mendelssohn & Steinitz, 1943) (Alytidae, Discoglossinae)), that has been recently rediscovered (Biton et al. 2013). However, there are still some species not recorded since the Hula drainage, for example, the beetle *Galerucellanymphaeae* (Furth 1993) and species of water beetles, mosquitoes, midges, dragonflies, giant water bugs, etc. (Dimentman et al. 1992). Before the drainage of the Hula, *Sparganiumerectum*, the food plant of *D.bicolora*, was found commonly along the western and eastern edges of the Hula Lake and Swamps but virtually disappeared after the drainage and is currently considered a protected plant in Israel. Historical information about the flora of the Hula can be found in Zohary and Orshansky (1947) and Zohary (1966).

Much of the history of this project was provided in two previous publications by the senior author (Furth 1977, 1993) and will not be repeated here. Many other historical aspects of the Hula region can be found in Paz (1976) and Dimentman et al. (1992). In Furth (1977, 1993) this species of *Donacia* was referred to as *D.bicolor* Zschach in error. *Donaciabicolora* has been recorded from most of eastern and western Europe, Iran, and Turkey (Löbl and Smetana 2010), making the Hula the most southern limit of distribution for this species, also true for many other taxa. It has been recorded in much of Europe as feeding on *Sparganiumerectum* (Mohr 1966). In Furth (1977) this host plant was mistakenly referred to as *S.neglectum*, but the error was corrected (Furth 1993).

The distributions of *Donacia* species recorded nearest to Israel are from Löbl and Smetana (2010), while the records confirmed by Geiser and Jäch (2021) are indicated by an asterisk (*). *Donaciabicolora* was also recorded from Israel by Furth (1977, 1993):

*D.bicolora*: Iran; Israel; Turkey; Albania*; Serbia*; Bosnia-Herzegovina*; Montenegro*

*D.marginata*: Iran; Israel; Greece: Turkey; Morocco; Bosnia-Herzegovina*; Serbia*

*D.simplex* Fabricius, 1775: Turkey; Algeria; Morocco; Croatia; Bulgaria; Serbia*; Iran*; Syria (Anti-Lebanon mountains)*

*D.tomentosa* Ahrens, 1810: Croatia*; Greece*; Iran; Israel*

Historically, specimens identified as *Donaciabicolora* documented in the entomology collection at the Steinhardt Museum of Natural History (SMNH TAU) (except as noted) are as follows (exact label data): Palestine, Hulata, 29 March 1940, Bytinski-Salz; Palestine, July 1940, Bytinski-Salz; Palestine, Binyamina, Kabarah, July 1940 Bytinski-Salz; Hula, 20 April 1941, leg. Steinitz et al. [larva, ex Hebrew University collections]; Hulatah, Palestine, 29 March 1942, Bytinski-Salz; Palestine, Hula 29 March 1942, Bytinski-Salz; Hula, 1 April 1942; Palestine, Hula 8 April 1945; Palestine, Huleh 25 April 1945, leg. Bytinski-Salz, on *Sparganium*. Haifa District 9 April 1945 [this record is from the Natural History Museum, London [NHM]), E. Geiser, pers. comm. 2022].

Because of the more recent records in SMNH TAU collection, the senior author began to investigate the potential that *D.bicolora* survived the drainage of the Hula Lake and Swamps.

## ﻿Material and methods

**Figure 3.**
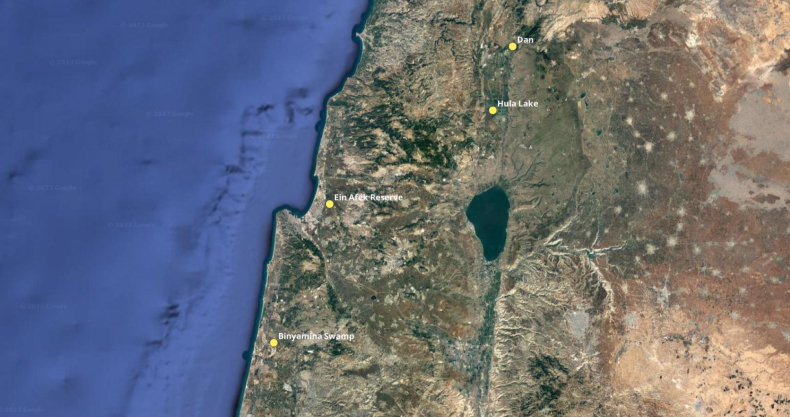
Map of Israel showing locations of Hula Lake, Kibbutz Dan, Ahu Binyamina (as Binyamina swamp), and Ein Afek.

For this study Israeli historical specimens of *Donacia* were examined from Steinhardt Museum of Natural History, Tel Aviv University, Israel (**SMNH TAU**). Between 2011 and 2022 the first author and Ariel “Laibale” Leonid Friedman collected regularly at the Dan River tributary behind the Bet Ussishkin Nature Museum belonging to Kibbutz Dan. This site was chosen because of indications in relatively recent history of the rediscovery of this *Donacia* based on specimens at SMNH TAU from 1993 and 2009 (see Results). Other collecting sites visited were Hula Lake Park, Kibbutz Dan, Ein Afeq, Binyamina swamps (Fig. 3), as well as some other potential sites such as Ein Nymphit, Baniass, and Granot Hadera canal (see Results for exact dates of visits).

Based on the 2018 information about the flora in the Israel Nature Protection and National Parks Authority databases (Y. Malihi, pers. comm. 2018) the senior author visited the following national parks in search of populations of *Sparganiumerectum* and *Donaciabicolora*: Hula Lake Preserve, Ahu Binyamina, Ein Nymphit, Ein Afeq, and Baniass.

Recently *Donacia* specimens were collected at Kibbutz Dan by sweep netting and then either placed directly into 95% ethanol or put into 95% ethanol shortly after collecting to preserve them for molecular analyses. Specimens used for molecular analyses were collected on 11/12 March 2013. Moreover, three *D.bicolora* specimens from the entomology collections of NHM collected in Ukraine were also included in this study as reference of precise identity for the molecular identification of Israel specimens. Some specimens were also pinned as vouchers for the museum collections at SMNH TAU. A photo of the lateral view in copula of this species dated 11/12 March 2013 was also sent to an expert on *Donacia* (Dr. Elisabeth Geiser, Salzburg).

DNA was extracted from three specimens of *D.bicolora* collected in Ukraine (collecting information: Ukraine - Volynska Oblast’, Shatsky District, Pischa vill., fishing ponds, 161 m, 51°35'53.8"N, 23°46'13.3"E 26.V.2019, K. Matsumoto leg., NHM(E) 2019-91) and from two *Donacia* sp. specimens from Israel (Israel - Upper Galilee, Kibbutz Dan, 12.III.2013, leg. D.G. Furth). Specimens processing took place at the laboratories of University of Milan, Italy. Non-destructive DNA extraction was performed from the whole insect body using Qiagen DNeasy Blood and Tissue Kit (Qiagen, Hilden, Germany) following the method described in Magoga et al. (2016). After DNA extraction, the voucher specimens were dry mounted on pins.

A 658 bp region of the mitochondrial cytochrome oxidase subunit 1 (COI) was amplified by PCR using the barcode primers LCO1490/HCO219821 (Folmer et al 1994). PCRs were performed in a volume of 25 μL reaction mix containing 1× GoTaq reaction Buffer (10 mM Tris-HCl at pH 8.3, 50 mM KCl and 1.5 mM MgCl_2_), 0.2 mM of each deoxynucleoside triphosphate, 0.5 pmol of each primer, 0.3 U of GoTaq DNA Polymerase, and 10/20 ng of template DNA. The adopted thermal protocol was that described in Montagna et al. (2013). Positive amplicons were directly sequenced on both strands using Sanger sequencing (Microsynth, Balgach, Switzerland). Consensus sequences were obtained by editing electropherograms using Geneious R8 (Biomatters Ltd., Auckland, New Zealand). The presence of an open reading frame was verified using the on-line tool EMBOSS Transeq (http://www.ebi.ac.uk/Tools/st/emboss_transeq/).

The obtained COI sequences were matched with those available in GenBank and in BOLD (Ratnasingham and Hebert 2007) databases through a Basic Local Alignment Search Tool analysis (BLAST, default parameters; BLAST: http://www.ncbi.nlm.nih.gov/BLAST; Altschul et al. 1990) and BOLD identification engine, respectively. Newly developed sequences were registered in BOLD system (BOLD IDs: MEDLB961-23 to MEDLB65-23).

All sequences of *Donacia* publicly available in BOLD were retrieved (March 2022; Suppl. material 1). Sequences derived from contamination were discarded. The remaining sequences were aligned at codon level using MUSCLE (Edgar 2004) together with the sequences developed in this study. Sequences with length < 400 bp were removed and haplotypes were reduced using R software (R v. 3.5.2; R Core Team 2020). The obtained dataset (91 sequences belonging to 26 species) was used for estimating K2P pairwise nucleotide distances between sequences using the R package ape (Popescu et al. 2012) and for the COI dendrogram inference. Prior to the dendrogram inference, the best nucleotide substitution model was estimated using jModelTest 2 (Darriba et al. 2012) and selected according to Akaike Information Criterion (AIC) (Akaike 1974). Maximum likelihood phylogenetic inferences were performed using PhyML version 3.0 (Guindon et al. 2010) with the following options: evolutionary nucleotide substitution model as obtained by model selection procedure (GTR + I + G); the best of NNI and SPR tree searching operation; approximate likelihood ratio test as node support (aLRT; Anisimova et al. 2011). A COI sequence of *Plateumarisbraccata* (Scopoli) was used as outgroup. A subset of the sequences retrieved from BOLD (15 species) aligned with the sequences generated in this study was used to infer a minimum-spanning haplotype network (Bandelt et al. 1999) with PopART software (Leigh and Bryant 2015).

## ﻿Results

### ﻿Museum specimens

**Figure 4.**
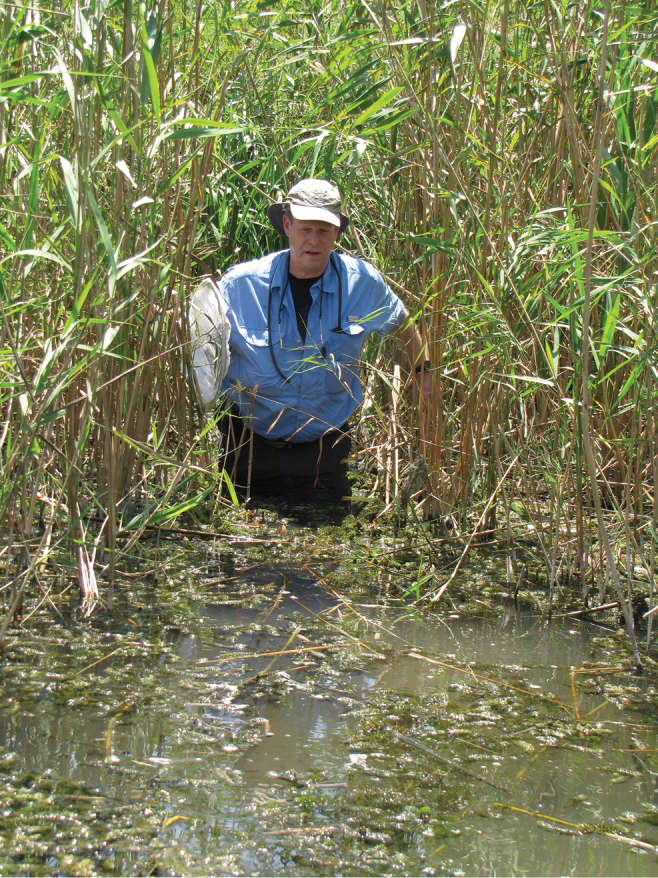
D. Furth searching for *Sparganiumerectum* and *Donaciasimplex* in the Hula Preserve in 2011 (photograph by Z. Yanai).

**Figure 5.**
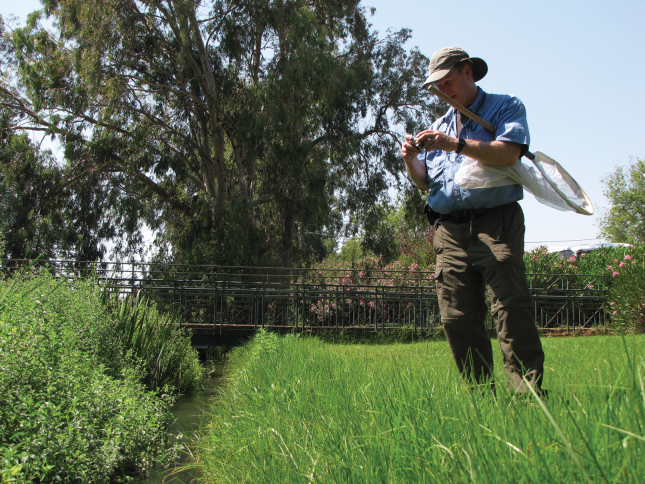
D. Furth searching for *Sparganiumerectum* and *Donaciasimplex* at Kibbutz Dan (Bet Ussishkin) in 2011 (photograph by Z. Yanai)

Relatively recent specimens collected in Israel and identified as *Donaciabicolora* were found within the entomology collections of SMNH TAU. These few specimens were as follows: Hula Lake Preserve, 1 July 1993, collected by V. Chikatunov (2 specimens); and Dan, 33°14'N, 35°39'E, 19 May 2009, collected by L. Goren (1 specimen). Because of these records, multiple field trips were made to the Kibbutz Dan (Dan River tributary = Bet Ussishkin) and to the Hula Preserve as well as other sites with either historical records of *D.bicolora* or those suspected of having populations of its food plant *Sparganiumerectum*, as follows (see Figs 4, 5).

### ﻿Collected material (in chronological order)

13 July 2011, the Hula Lake Preserve and Kibbutz Dan (Dan River tributary = Bet Ussishkin), *Sparganiumerectum* was swept by D. Furth (DF), L. Friedman (LF), Z. Yanai (ZY), but no *Donacia* were found.

**Figure 6.**
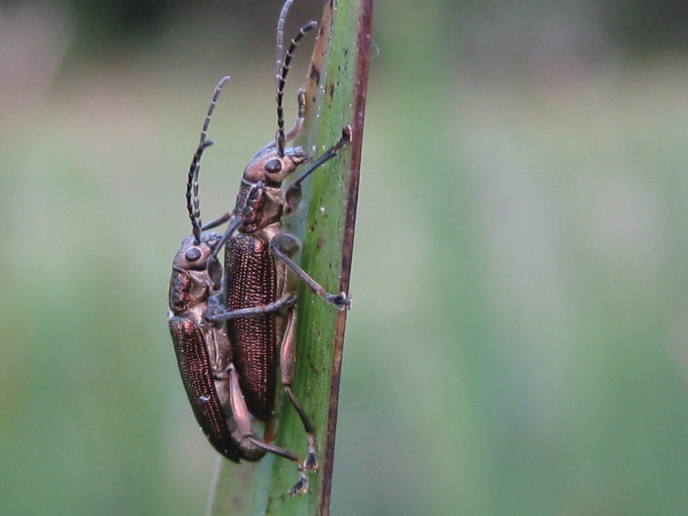
*Donaciasimplex* adults in copula (photograph by D. Furth).

**Figure 7.**
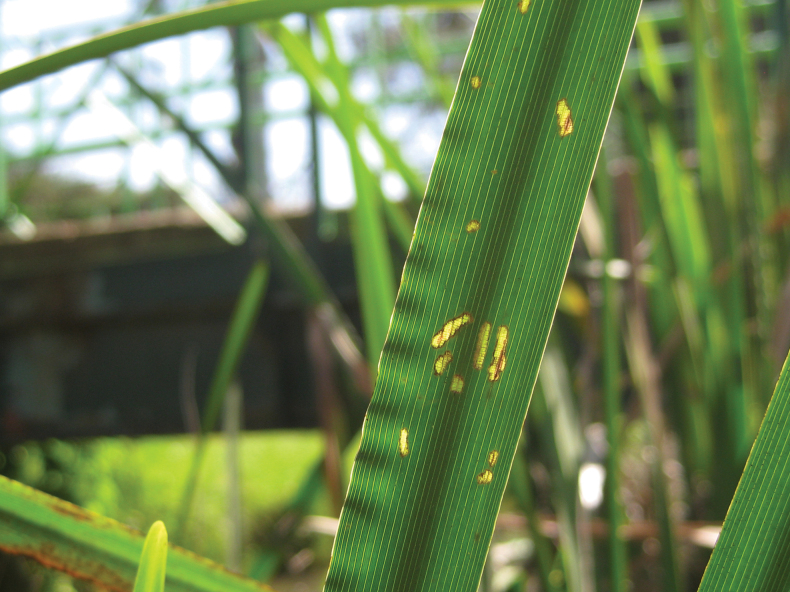
*Donaciasimplex* adult damage on leaf of *Sparganiumerectum* (photograph by D. Furth).

**Figure 8.**
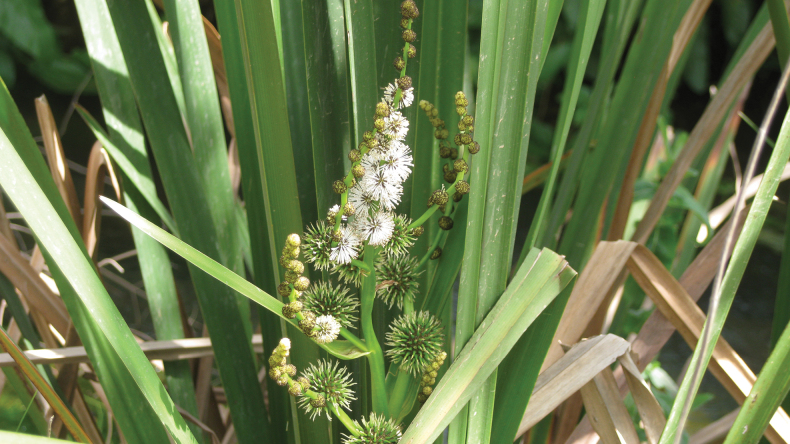
*Sparganiumerectum* flowers (photograph by D. Furth).

11–12 March 2013 DF collected many *Donacia* at the Bet Ussishkin location, including mating pairs (see Figs 6–8).

1 April 2014 DF and ZY visited Ahu Binyamina (32.501465°N, 34.946319°E), no *Sparganium* were found, no *Donacia* collected.

1 April 2014 DF and ZY visited the Hadera Granot canal (32.449819°N, 34.939678°E), a large population of *Sparganium* was found, but no *Donacia* collected.

26 March 2015 DF checked Bet Ussishkin, *Donacia* leaf damage, no *Donacia*.

28 April 2015 DF collected at Dan, no *Donacia*.

3 March 2016, 1 May 2016, and 20 February 2018 a few *Donacia* were collected by LF at Dan stream (Bet Ussishkin).

8 March 2017 DF checked Ahu Binyamina, no *Donacia* found.

14 March 2017 DF checked Granot Canal, Hadera (32.26.979N, 34.56.384E, 12 m), no *Donacia* found.

15 March 2017 Bet Ussishkin, DF and LF checked, no *Donacia* found or plant damage.

20 February 2018 LF collected 3 *Donacia* at Bet Ussishkin.

13 March 2018 DF collected *Donacia* at Bet Ussishkin but none at Hula Park or Baniass Park.

15 March 2018 DF and LF collected at Ein Afek (no *Sparganium* found) and at Ein Nymphit (one *Sparganium* found), but neither location produced *Donacia*.

27 February 2020 LF collected a few *Donacia* at Bet Ussishkin.

16 March 2020 LF collected a few *Donacia* at Bet Ussishkin.

18 March 2022 DF checked *Sparganium* at Bet Ussishkin, no *Donacia* found.

### ﻿Molecular identification

The COI sequences obtained from the three specimens collected in Ukraine belong to two different haplotypes, while both specimens from Israel shared the same haplotype.

**Figure 9.**
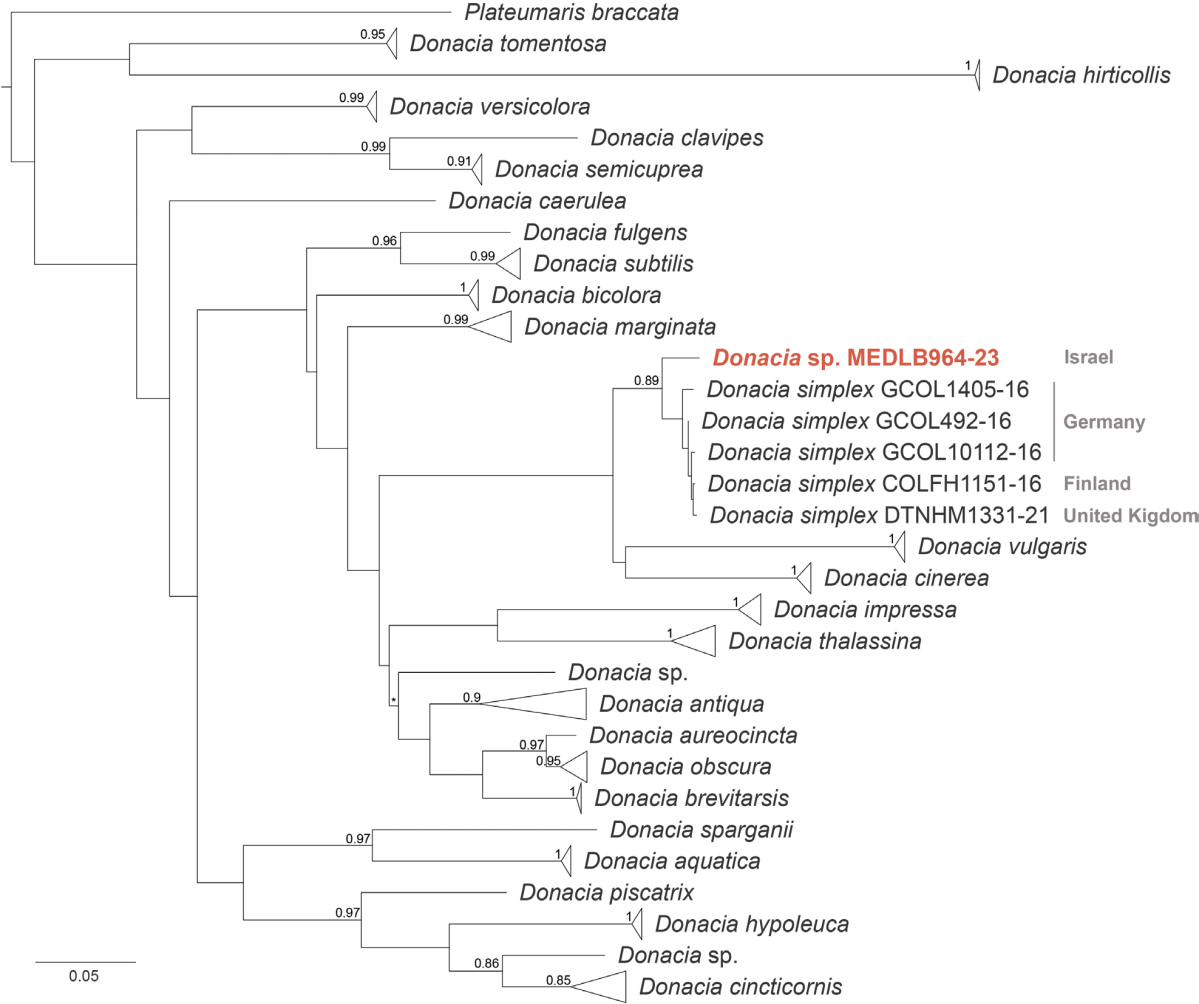
Maximum likelihood dendrogram of the genus *Donacia* inferred using COI gene nucleotide sequences. Terminal nodes are visually collapsed to species level, except in the case of *D.simplex* clade. *Donacia* sp. collected in Israel is indicated in red. Collection countries of the specimens falling in the *D.simplex* clade are indicated in grey. The tree scale bar indicates the distance in nucleotide substitutions per site. The aLRT values are reported on nodes; * represents aLRT values < 0.70 (created by M. Montagna and G. Magoga).

**Figure 10.**
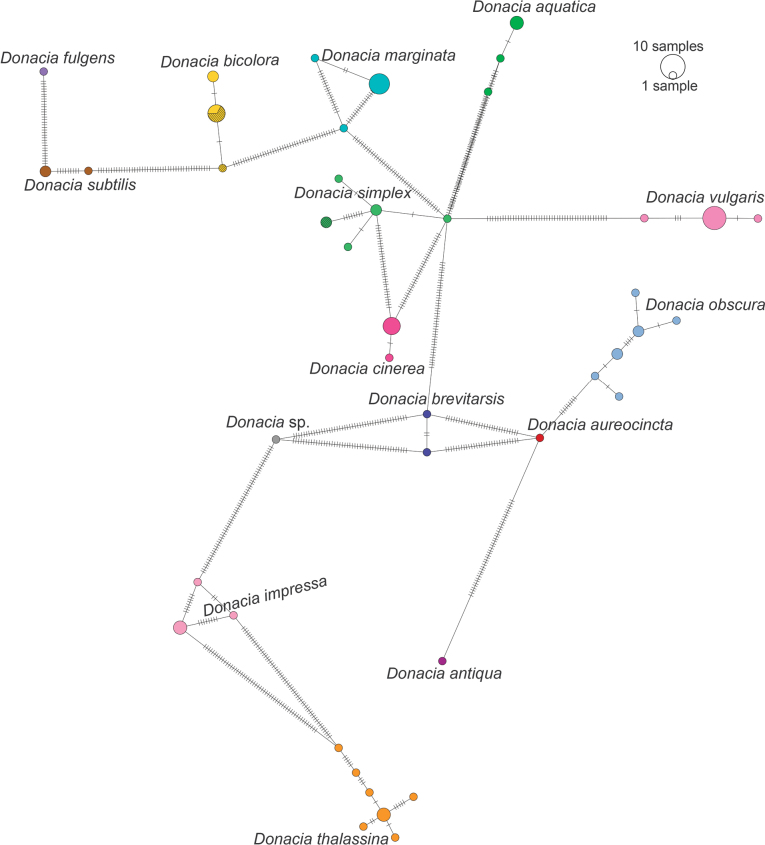
Minimum Spanning Haplotype Network of *Donacia* inferred from COI gene nucleotide sequences. Each color represents a species. The diameter of the circle is proportional to the abundance of the haplotypes, vertical lines on edges represent the nucleotide substitutions between haplotypes. Diagonal lines on yellow and green circles identify haplotypes of Ukrainian and Israeli specimens (created by M. Montagna and G. Magoga).

The individuals of *D.bicolora* collected in Ukraine showed an identity between 99.7% and 100% (e-value < 1×10^–20^) with sequences of *D.bicolora* collected in Finland (BLAST analysis) and a similarity between 99.5% and 100% with sequences of *D.bicolora* collected in Finland and Norway (BOLD identification engine analysis). For the specimens collected in Israel, an identity between 96.5% and 96.8% (e-value < 1×10^–20^) was observed with sequences of *D.simplex* collected in Finland and Germany using BLAST; using BOLD, a similarity between 97.3% and 97.7% was observed with the same sequences. A nucleotide distance of ~ 13% was estimated between the sequences of *D.bicolora* from Ukraine and those of *Donacia* from Israel generated in this study. In the maximum likelihood tree inferred from the alignment of *Donacia*COI sequences, *Donacia* from Ukraine clustered in a monophyletic group with other *D.bicolora* (aLRT = 1) (Fig. 9), whereas the individuals from Israel grouped with *D.simplex* from Germany, Finland, and United Kingdom (aLRT of 0.89) (Fig. 9). In the haplotype network, the same clusters were observed (Fig. 10).

### ﻿Note

After examination of the photograph (lateral view) of a copulating pair of this species (see Fig. 6) from the Kibbutz Dan population collected on 11/12 March 2013, Dr. E. Geiser was not able to definitively identify it as *D.bicolora* or *D.simplex* based on its morphology (E. Geiser, pers. comm. 2023). Dr. Geiser confirmed that the photograph in Furth (1977: fig. 3) was indeed *D.bicolora* but the specimen in that photograph was not from Israel, but from the entomology collections at the Museum of Comparative Zoology, Harvard University, only as a representative of *D.bicolora*.

## ﻿Discussion

The nucleotide distance comparison between the sequences of *D.bicolora* (whose identity was further confirmed through the molecular analyses) from Ukraine with those of the *Donacia* specimens from Israel, as well as the *Donacia* dendrogram and the haplotype network analyses, suggest that the *Donacia* specimens collected in Israel do not belong to *D.bicolora*. The Israeli *Donacia* is closer to *D.simplex*, having a sequence identity/similarity of ~ 97% with *D.simplex* sequences publicly available in the reference databases. The ~ 3% nucleotide distance value estimated between *D.simplex* public COI sequences and those of *Donacia* from Israel generated in this study is higher than the optimal threshold for the molecular identification of Donaciinae identified in Magoga et al. (2018). However, from the haplotype network, we can observe that the number of nucleotide substitutions elapsing between Israeli *Donacia* and *D.simplex* from Finland and Germany is comparable to the one observed between the different haplotypes of other species (e.g., *D.marginata* Hoppe and *D.impressa* Paykull) (Fig. 10). This evidence suggests that this species of *Donacia* from Israel is *D.simplex*, and that the nucleotide distance observed is likely related to the geographic distance between the considered populations. The clusters found on the *Donacia*COI dendrogram further confirmed these molecular identifications (Fig. 9).

The results obtained in this study by integrating various sources of evidence (analyses of historical collections, faunal surveys, the morphological and molecular analyses) have revealed that the species present in Israel that has been historically referred to as *D.bicolora* (in SMNH TAU collections; Furth (1977, 1993)) is actually *D.simplex*. Previous identifications of this species, especially within the collections in Israel, were not verified by *Donacia* experts thus creating a misunderstanding that protracted over time.

Based on morphological observations, especially the photograph in Furth (1977: fig. 3, as a representative of *D.bicolora* and not from Israel), and the fact that the species in Fig. 6 cannot definitively be identified by morphology, Dr. E. Geiser still has concerns as to the identity of this single species from the Hula Valley and she feels there may be some discrepancy between the molecular and morphological identity of this single species of *Donacia* (*D.simplex* or *D.bicolora*, respectively). Therefore, subsequent molecular and morphological research about this will be conducted. Nevertheless, it is a single species from the Hula Valley and the principles of its rediscovery and conservation will not change no matter what the taxonomic nomenclature finally reveals.

Even though Donaciinae species are globally primarily Holarctic, it is probable that other species of *Donacia* that are possibly cryptic may be discovered or rediscovered in the biogeographically diverse biotopes of Israel in the future, such as *D.marginata* (Furth, 1993) or *Donaciatomentosa* (Geiser and Jäch 2021).

**Figure 11.**
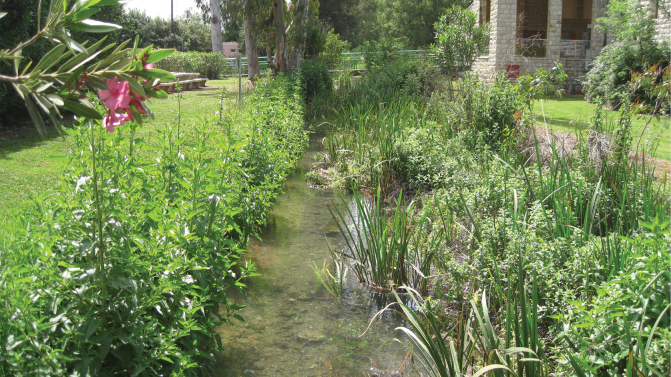
Kibbutz Dan, Bet Ussishkin canal with *Sparganiumerectum* (photograph by D. Furth).

**Figure 12.**
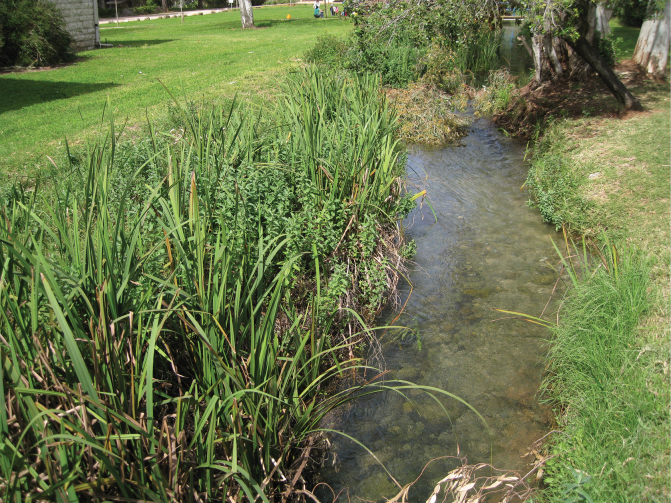
Kibbutz Dan, Bet Ussishkin canal cleaned on one side (photograph by D. Furth).

**Figure 13.**
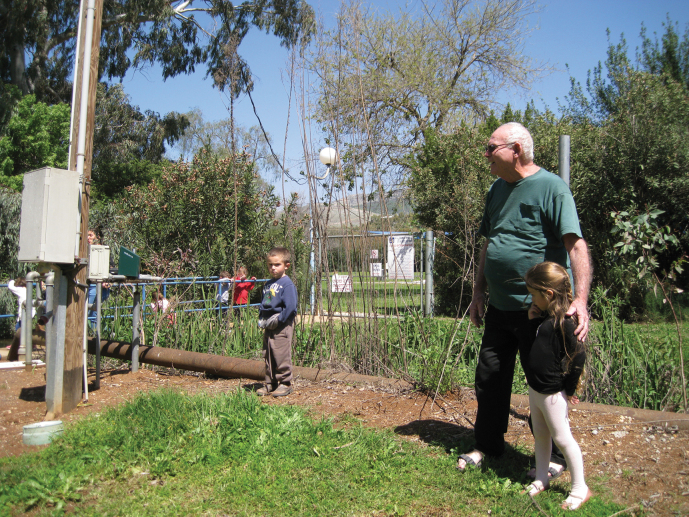
Yossi Levari with two of his grandchildren, near the Bet Ussishkin canal, Kibbutz Dan (photograph by D. Furth with permission from Rachel Levari).

Based on the results of this study, the most viable re-discovered population of *D.simplex* is present at Kibbutz Dan. It may also be present in much smaller numbers in the Hula Lake Preserve/National Park based on the 1993 record mentioned above in the Results. This establishes the Israeli populations of *D.simplex* as the southernmost population of this species. However, the Kibbutz Dan population is endangered because the primary population of the food plant (*S.erectum*) is at risk because the water is directed via this canal to the Kibbutz fishponds (Fig. 11): in order to allow the water to flow more easily, Kibbutz workers have been clearing all plants from this canal each year (Fig. 12). The late Yossi Levari (a long-time Kibbutz member, botanist, and former director of the Kibbutz Dan Bet Ussishkin Museum) arranged an informal agreement with the Kibbutz fisheries to allow *S.erectum* plants to remain and grow on the south side of the canal behind the Museum, thus allowing the population of *D.simplex* to flourish. However, Mr. Levari passed away in 2020 and the informal agreement with the Kibbutz fisheries could change at any time without a more formal written agreement. This publication is dedicated to Mr. Levari (Fig. 13).

**Figure 14.**
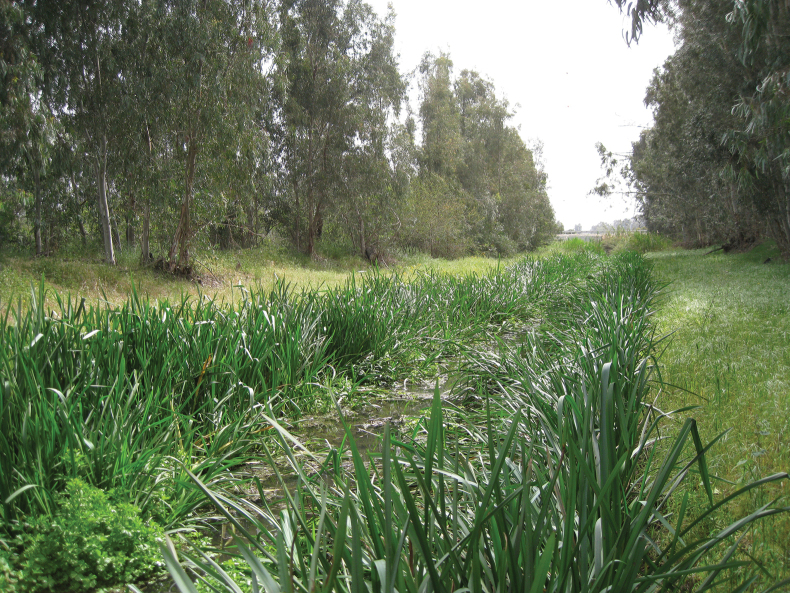
Hadera Granot canal, full of *Sparganiumerectum* (photograph by D. Furth).

This study has revealed the necessity to try to conserve any populations of *D.simplex* as well as its food plant *S.erectum*. Although there is currently no endangered and threatened list for insects in Israel, there is a plan to create something similar via a website (Z. Yanai, pers. comm. 2023). When such a list is compiled in the future, this species (*D.simplex*) should be included. Fortunately, its food plant, *S.erectum*, is on the Red List of Endangered Plants of Israel (Y. Malihi, pers. comm. 2018). A future goal is to try to re-introduce *Donaciasimplex* to locations where it was historically recorded, e.g., Binyamina (Ahu Binyamina) as well as other parks or locations that support its food plant (*S.erectum*), e.g., Ein Afek. Another such potential re-introduction location is the Granot Canal near Hadera (Fig. 14) where there is a large population of *S.erectum*; however, such locations that are not protected as in a park could be decimated by local authorities for road enhancement, construction of housing, and in Granot Canal, agriculture is a threat due to pollution from pesticide run off from adjacent fields. Therefore, the optimum reintroduction locations should be in protected areas.

## ﻿Conclusion

The Israeli populations of this semi-aquatic Leaf Beetle have been historically referred to as *D.bicolora*, but the molecular analysis presented here demonstrates that it belongs to *D.simplex*. The historical records from museum specimens, primarily in SMNH TAU, indicated that this species may have been extirpated from the region because of the drainage of the Hula Lake and Swamps in the 1950s, because its food plant *S.erectum* was also severely restricted due the drainage. However, the evidence presented in this study demonstrates that *D.simplex* has survived in very limited populations and should be preserved to enable it to flourish once again.

## ﻿Additional information

### Author ORCIDs

David G. Furth https://orcid.org/0000-0001-6559-1209

Matteo Montagna https://orcid.org/0000-0003-4465-7146

Giulia Magoga https://orcid.org/0000-0002-0662-5840

### Data availability

All of the data that support the findings of this study are available in the main text or Suppl. material 1.

## Supplementary materials

Supplementary material 1 Sequences retrieved from BOLD system for performing the analyses of this study https://doi.org/10.3897/zookeys.1177.101498.suppl1 Data type table (Excel spreadsheet) https://binary.pensoft.net/file/898724This dataset is made available under the Open Database License (http://opendatacommons.org/licenses/odbl/1.0/). The Open Database License (ODbL) is a license agreement intended to allow users to freely share, modify, and use this Dataset while maintaining this same freedom for others, provided that the original source and author(s) are credited.David G. Furth, Matteo Montagna, Giulia Magoga
